# A Dynamic Finite Element Analysis of Human Foot Complex in the Sagittal Plane during Level Walking

**DOI:** 10.1371/journal.pone.0079424

**Published:** 2013-11-11

**Authors:** Zhihui Qian, Lei Ren, Yun Ding, John R. Hutchinson, Luquan Ren

**Affiliations:** 1 Key Laboratory of Bionic Engineering, Jilin University, Changchun, P.R. China; 2 School of Mechanical, Aerospace and Civil Engineering, University of Manchester, Manchester, United Kingdom; 3 Editorial Department of Journal of Bionic Engineering, Jilin University, Changchun, P.R. China; 4 Structure and Motion Laboratory, The Royal Veterinary College, University of London, Hatfield, United Kingdom; German Cancer Research Center, Germany

## Abstract

The objective of this study is to develop a computational framework for investigating the dynamic behavior and the internal loading conditions of the human foot complex during locomotion. A subject-specific dynamic finite element model in the sagittal plane was constructed based on anatomical structures segmented from medical CT scan images. Three-dimensional gait measurements were conducted to support and validate the model. Ankle joint forces and moment derived from gait measurements were used to drive the model. Explicit finite element simulations were conducted, covering the entire stance phase from heel-strike impact to toe-off. The predicted ground reaction forces, center of pressure, foot bone motions and plantar surface pressure showed reasonably good agreement with the gait measurement data over most of the stance phase. The prediction discrepancies can be explained by the assumptions and limitations of the model. Our analysis showed that a dynamic FE simulation can improve the prediction accuracy in the peak plantar pressures at some parts of the foot complex by 10%–33% compared to a quasi-static FE simulation. However, to simplify the costly explicit FE simulation, the proposed model is confined only to the sagittal plane and has a simplified representation of foot structure. The dynamic finite element foot model proposed in this study would provide a useful tool for future extension to a fully muscle-driven dynamic three-dimensional model with detailed representation of all major anatomical structures, in order to investigate the structural dynamics of the human foot musculoskeletal system during normal or even pathological functioning.

## Introduction

The human foot is a very complex structure comprising numerous bones, muscles, ligaments, synovial joints and other tissues. As the only body component in contact with the ground, it plays multiple crucial roles in attenuating ground impacts, supporting against gravity, maintaining locomotor stability and transmitting or generating propulsive power during locomotion [1]–[3]. Over recent decades, a large number of experimental and computer simulation studies have investigated how the human foot complex functions in locomotion [4]–[9].

Of the numerous mathematical modelling approaches used for analyzing biomechanical foot functions, finite element (FE) analysis offers a powerful tool to assess the internal loading conditions of the foot musculoskeletal structure during human locomotion [5], [10]. It can provide valuable estimates of stress and strain distributions in the foot bones and soft tissues, which are usually not measurable in vivo. Lemmon et al. [11] used a two dimensional (2D) FE foot model to study the effects of the shoe insole on therapeutic footwear biomechanics. A stress analysis study was conducted on both normal and neuropathic feet using a 2D foot FE model derived from a lateral X-ray image [12]. Jacob et al. [13], [14] conducted quasi-static analyses of different gait phases using a three-dimensional (3D) foot FE model to investigate tarsal bone degeneration in diabetes and Hansen's disease. A 3D FE foot model with detailed bone and soft tissue representations was constructed by Gefen et al. [5], in which quasi-static analyses were performed for six different instantaneous gait phases. A subject-specific 3D FE model including detailed foot structure geometry and non-linear material properties was also developed [15]–[17] with demonstrated potential for medical applications. Wu [18] built a 2D model to investigate the foot bone and muscle stresses resulting from plantar fasciotomy and plantar ligament injuries. Recently, a 2D FE foot model was integrated with a multi-body musculoskeletal model to simulate tissue mechanics and musculoskeletal movements simultaneously [19], [20], in which prescribed kinematic boundary conditions were used to drive the foot model.

Due to the complexity of the human foot musculoskeletal structure, most previous FE models involved over-simplified loading and boundary conditions. Moreover, the analyses were static or quasi-static in nature. Yarnitzky et al. [21] developed a two dimensional hierarchal foot model, which coupled an analytical rigid body foot model with a local FE analysis, to study the internal deformations and stresses of heel and metatarsal head regions in real-time. In the rigid body model, simplified bone structure was used, by assuming to have a rotational axis in the center of cuneiform, to provide loading and boundary conditions for the FE analysis, which was quasi-static in nature. So far, there are very few foot modelling studies using explicit FE simulation [22]–[24]. One such investigation into foot locomotor biomechanics is that by Dai et al. [25], which examined the effect of sock wearing on plantar pressures, rather than foot-ground interactions. In their study, the load was assumed to be constant throughout the stance phase and kinematic constraints were used to define the foot motion. Simplified loading and boundary conditions and excessive constraints may lead to poor prediction of foot loading conditions during natural human movements. As a result, although quasi-static foot function has been reasonably represented, modelling and simulation studies to date have illuminated only limited details about the more realistic, dynamic response of the foot complex during human locomotion.

The objective of this study is to develop a subject-specific dynamic FE human foot model based on individualized medical imaging data and loading conditions derived from gait measurements on the same subject. No prescribed kinematic conditions are used to constrain the foot motions, which allows for the representation of natural foot movements during human locomotion. The model's validity was rigorously tested by comparing the predicted foot bone motions, ground reaction forces, center of pressure (CoP) and plantar pressure distributions with the measured data.

## Materials and Methods

### Ethics Statement

This study was approved by the Institutional Review Board Committee of Jilin University, Changchun, China, and the subject gave his written informed consent (as outlined in PLOS consent form) to participate in the CT scanning and gait measurements.

### Finite Element Foot Modeling

**Figure 1:**
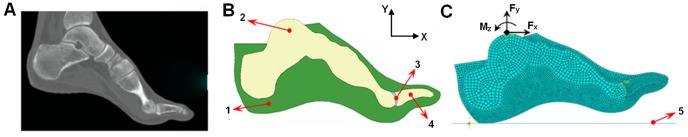
The 2D finite element modelling of the human foot complex. (A) The foot CT image along the first ray. (B) The 2D foot geometric model based on the CT image. (C) The 2D finite element foot model. (1. soft tissue, 2. bone structure except phalanges, 3. cartilage, 4. phalangeal bones, 5. ground surface)

The foot geometric model was constructed from medical CT images (Lightspeed 16, General Electric Company, Fairfield, U.S.A.), which were obtained by scanning the right foot of a healthy male subject (age: 27 yrs, weight: 75 kg; no history of lower limb injury or foot abnormalities) in the neutral unloaded position with a 1.5 mm slice interval, 220 mA and 120 kV. A 2D cross section in the sagittal plane along the first ray of the foot was taken to construct the geometric model (similar to [19], [20]) using the SolidWorks software (Dassault Systèmes SolidWorks Corp., U.S.A.). The 2D planar model, as shown in Fig. 1, consisted of four segments. The bones other than the phalanges were considered as one segment. All the phalanges were represented as one separate part, which could move freely with respect to the metatarsal bones. The other two segments were soft tissues wrapping around the foot bones and the cartilages between the phalanges and the metatarsal bones. The geometric model was then imported into and assembled in the FE software package ABAQUS (Simulia, Providence, U.S.A.).

An analytical rigid flat plate was used to simulate the ground support. Interaction between the foot and the ground was modeled as a kinematic contact with a frictional coefficient of 0.6 [16]. The kinematic constraint method uses a predictor/corrector algorithm in each time increment to strictly impose contact constraints (no substrate penetration is allowed) and to conserve momentum [26]. The constraints are imposed on the global equations by a transformation of the nodal displacement components of the slave nodes along the contact interface. In the corrector phase, the depth of each slave node's penetration, its associated mass, and the time increment are used to calculate the resistant force required to prevent penetration. In the horizontal direction, the finite sliding method, allowing for arbitrary separation, sliding and rotation of the contact surfaces, was employed [26]. The bones and soft tissues were meshed using a total number of 4259 quadrilateral elements, which was determined through a convergence analysis by gradually increasing the mesh density until the deviations in the estimated stresses reached <5% [5]. The soft tissue of the foot was assumed to be homogeneous, isotropic and linearly elastic [27]. A Rayleigh material damping coefficient (alpha) 6.7 was defined to represent the viscous behavior of the foot soft tissue in ABAQUS [26]. The damping coefficient was determined using a trial and error process to minimise the ground reaction force oscillations generated after the heel strike in the simulation. The metatarsal-phalangeal joint in the sagittal plane was connected using hyperelastic cartilages [18]. The material properties of all structures modelled in this study are listed in Table 1, taken from the literature as cited. Plane stress section thickness was set to 60 mm, an approximate foot width of the subject, to achieve a reasonable foot mass in the simulation. This resulted in a total foot mass of 1.025 kg, about 1.37% of the subject's body mass, which is very close to that estimated using a cadaver-based anthropometric regression method [28].

**Table 1:** The material properties and element types used in the foot finite element model.

Component	Element type	Young's modulus (MPa)	Poisson's ratio	Mass density (kg/m^3^)	Reference
Soft tissue	quadrilateral	1.15	0.49	937.0	[27]
Bone structure	quadrilateral	7300	0.30	1500	[25]
Phalanges	quadrilateral	7300	0.30	1500	[25]
Cartilage	quadrilateral	10, Neo-Hookean hyperelastic	0.49,C_10_ = *E*/(4(1+*ν*))	2000	[18]

The vertical and horizontal forces and the net muscle moment for the ankle joint over the whole stance phase were calculated using the inverse dynamics method [28] based on the measured segmental motions and the ground reactions (see Gait Measurements in the next section). The time traces of these ankle joint forces and moment were then used as inputs to the FE model as the dynamic loading conditions by applying them to the middle of the talus bone as the only driving forces and moment. The ground was fixed and the foot was allowed to move freely with respect to the ground without any prescribed kinematic constraints. The initial conditions of the system, including the horizontal and vertical ankle joint velocities *v_x_*, *v_y_*, foot angle *θ_z_* and angular velocity *ω_z_*, were determined based on the measured motion data at heel-strike. The dynamic FE simulations were conducted using the ABAQUS/Explicit module to calculate the dynamic foot responses over the entire stance phase from heel-strike until toe-off. The simulation time period was set as identical to the gait measurement. The segmental inertial properties (mass, center of mass and moments of inertia) were automatically calculated by the ABAQUS software based on the model geometry and the material properties. The stability of the explicit dynamic simulation was verified by ensuring that the change of the total system energy of the foot complex was well within 1% after the whole simulation period.

### Gait Measurements

**Figure 2:**
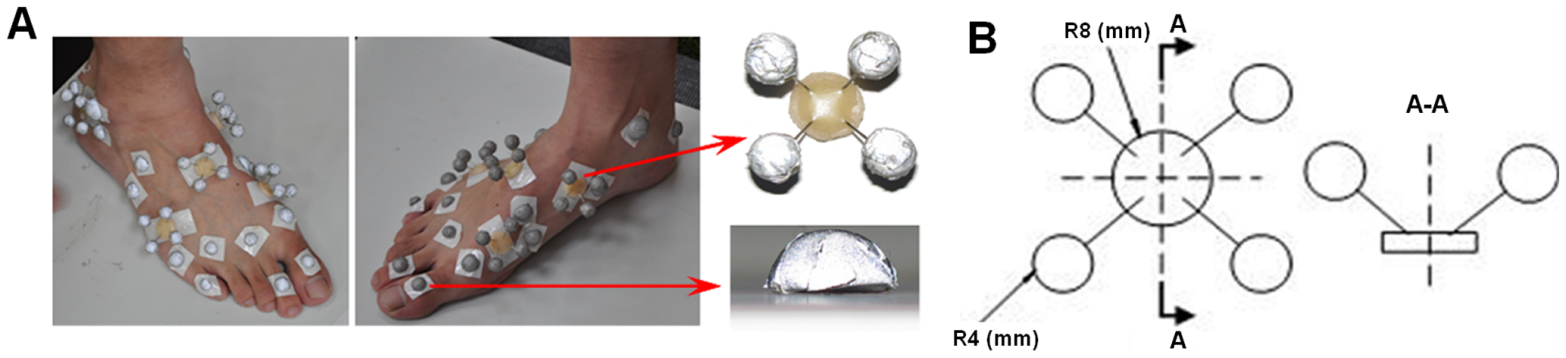
The infrared marker cluster system used in this study to capture 3D foot motions. (A)The foot was divided into five segments including hindfoot, midfoot, medial and lateral forefoot and toes. A set of thermal plastic plates, each carrying four infrared markers was mounted firmly on each segment to capture the segmental motions. A number of hemispherical infrared markers were also attached on the anatomical landmarks. (B) The configuration of the rigid marker cluster and the hemispherical marker.

Three-dimensional gait measurement was conducted to inform and validate the FE modelling. The same healthy male subject was used as in the modelling part of the study (see preceding section). A 12-camera infrared motion analysis system (Qualisys, Sweden) was used to capture the 3D motions of the lower limb segments and the foot segments at 150 Hz. A six force plate array (Kistler, Switzerland) was used to record the 3D ground reactions at 1000 Hz and a 1-meter-long pressure plate (RSscan, Belgium) was used for foot pressure distribution recording at 250 Hz. All three systems were digitally synchronized. A set of specially designed infrared reflective marker clusters was used (similar to [29]) to capture the delicate 3D multi-segment foot motions (see Fig. 2). The calibrated anatomical system technique [30] was used to determine the anatomical landmarks. The subject was instructed to walk barefoot at normal walking speed along a level indoor walkway. The trials were repeated ten times to ensure a representative gait pattern was obtained.

The measurement data were processed using GMAS software (the upgraded version of SMAS software), a MATLAB based software package for 3D kinematic and kinetic analysis of general biomechanical multi-body systems [28], [31]. The marker data were filtered using a low pass zero lag fourth-order Butterworth digital filter with a cutoff frequency of 6.0 Hz. The ankle joint forces and moment were calculated using inverse dynamics [28], and were used as the only muscular control variables for the foot FE model (see the FE Foot Modelling section).

### Error and sensitivity analysis

The prediction accuracy of the FE foot model was quantified using the root mean square error (RMSE) and the relative root mean square error (rRMSE) ([32], [33]) between the model predictions and the measurement data, which included the horizontal and vertical ground reaction forces, center of pressure, foot and phalanx rotation angles, and vertical pressures at heel and metatarsal regions over the whole stance phase. Sensitivity analysis was also conducted to investigate the effect of material properties, the frictional coefficient and the damping coefficient of the linear bulk viscosity on the model prediction results. The material properties analysis included four cases in which we changed the Young's modulus (*E*) of the linear elastic soft tissue by +20%, +10%, −10% and −20% from the baseline value (1.15 MPa), and also two different types of non-linear material property representations (Ogden hyperelastic based on [34] and polynomial hyperelastic based on [16]). The frictional coefficient analysis included four cases in which we changed the frictional coefficient of the foot-ground contact by +10%, +5%, −5% and −10% from the baseline value (0.6). Whereas, the damping coefficient *b* of the linear bulk viscosity was changed from its system default value of 0.06 to 2.0, 4.0, 6.0 and 8.0 respectively in the sensitivity analysis.

Additionally, in order to compare the difference between the static FE analysis and dynamic FE analysis, quasi-static FE simulations were conducted using the same FE foot model configuration defined in FE Foot Modeling section for the dynamic FE analysis. The quasi-static FE analysis was conducted at three representative instants of time over the gait cycle, i.e. just after the heel strike (0.03 second), around the middle stance (0.19 second) and just before the toe off (0.44 second). The calculated ankle joint forces and moment at each representative instant of time were used as the loading conditions for the static simulation of each case. The calculated peak plantar pressures in the heel, metatarsal and toe regions were then compared with the peak pressure values in the three regions obtained from the dynamic FE simulation at each simulated instant of time respectively.

## Results

**Figure 3:**
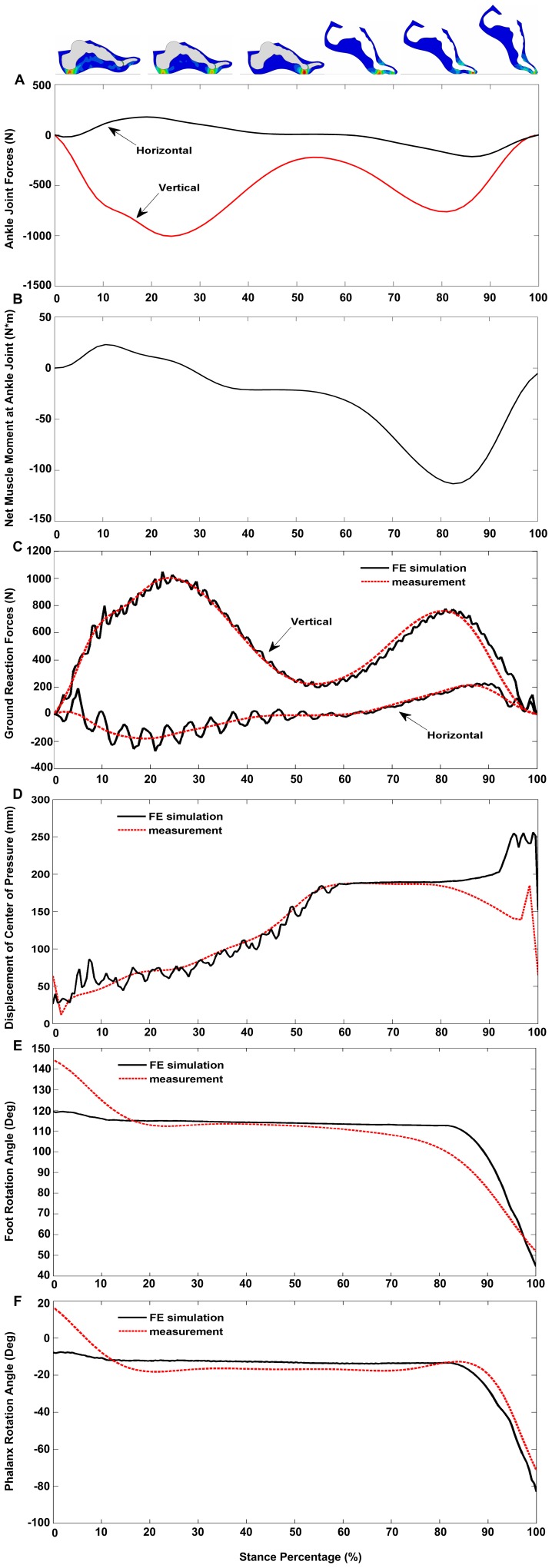
The FE model inputs and the FE simulation results over the whole stance phase compared with the measurement data. (A) Ankle joint force inputs. (B) Ankle moment input. (C, D, E and F) The FE simulation results compared with the measurement data.

The ankle joint forces and moment (see Fig. 3A and 3B) as well as the initial condition data from a representative gait cycle (walking speed 1.58ms^−1^) were used as inputs to the dynamic FE simulation. The obtained simulation results are shown in Fig. 3C to 3F. The predicted horizontal and vertical ground reaction forces over the stance phase are compared with the force plate data in Fig. 3C. The predicted ground reaction forces showed good agreements with the measured data in terms of the general trend with rRMSEs of 13% and 10% respectively (see Table 2). However, some apparent oscillations were observed especially in horizontal components in the early stance. Fig. 3D shows the predicted CoP trajectory over the stance phase compared with the measured force plate data. Although there are some small fluctuations during early stance, the predicted CoP shows a good agreement with the measurement data throughout most of the stance phase. The major discrepancy is in the late stance phase just before toe-off, where the FE model predicts a different CoP trend with a larger displacement than the measured data. Fig. 3E and 3F show the predicted foot rotation angle and phalanx rotation angle in the sagittal plane compared with the measured data. The model predictions match the measurement data well with relative RMSEs of 9.2% and 9.3% respectively, except for some discrepancies in the early stance phase.

**Table 2:** The result of material properties, frictional coefficient and damping coefficient sensitivity analyses of the foot finite element model.

Parameters^a^		F_x_		F_y_		CoP		FRA		PRA		HP		MP	
		RMSE(N)	rRMSE(%)	RMSE(N)	rRMSE(%)	RMSE(mm)	rRMSE(%)	RMSE(deg)	rRMSE(%)	RMSE(deg)	rRMSE(%)	RMSE(MPa)	rRMSE(%)	RMSE(MPa)	rRMSE(%)
Linear Elastic	+20%	54.11	10.86	115.02	10.64	0.048	23.34	8.01	9.87	7.45	9.41	0.035	16.55	0.054	21.30
	+10%	56.46	12.12	117.44	10.79	0.032	15.35	7.94	9.64	7.28	9.10	0.031	15.55	0.055	22.60
	0%	63.43	13.18	101.73	9.59	0.045	21.48	7.87	9.23	7.70	9.32	0.032	16.66	0.051	22.37
	−10%	55.43	11.62	128.86	11.45	0.048	23.20	8.08	10.05	7.23	9.29	0.036	18.16	0.050	22.10
	−20%	58.11	11.91	168.00	14.13	0.051	24.66	8.15	10.27	7.06	9.12	0.035	17.70	0.10	47.24
Hyper-elastic(Ogden)		71.98	15.01	257.32	24.54	0.051	22.53	31.27	15.85	22.28	14.86	0.050	34.07	0.055	31.28
Hyper-elastic(Polynomial)		76.68	14.29	254.08	23.54	0.041	17.66	23.23	14.69	21.47	14.66	0.040	24.96	0.067	35.38
Frictional Coefficient	+10%	41.38	9.41	45.81	4.51	0.026	13.31	8.47	11.25	7.28	10.01	0.035	18.80	0.043	20.92
	+5%	43.14	9.64	47.10	4.67	0.027	13.56	8.21	10.71	7.36	9.79	0.034	18.50	0.042	20.73
	−5%	34.85	8.35	41.33	4.07	0.012	7.51	17.06	34.26	12.16	25.43	0.033	18.33	0.054	26.51
	−10%	34.86	8.43	42.07	4.12	0.012	7.63	17.05	34.21	12.14	25.38	0.032	18.05	0.054	26.46
Damping coefficient (b)	2	86.57	18.51	282.53	27.98	0.034	17.72	10.03	12.21	7.42	10.07	0.073	31.21	0.074	30.92
	4	29.33	6.99	66.44	6.52	0.017	10.51	17.23	35.32	12.36	26.56	0.059	25.44	0.077	27.93
	6	35.84	8.39	35.73	3.56	0.016	10.51	17.08	34.25	12.17	25.56	0.061	26.35	0.069	25.71
	8	33.08	7.90	35.67	3.56	0.016	10.10	17.09	34.34	12.18	25.63	0.061	26.44	0.069	25.85

a RMSE  =  root mean square error, rRMSE  =  relative root mean square error, F_x_  =  horizontal component of ground reaction force, F_y_  =  vertical component of ground reaction force, CoP  =  displacement of center of pressure, FRA  =  foot rotation angle, PRA  =  phalanx rotation angle, HP  =  plantar pressure in the heel medial region, MP  =  plantar pressure in the 1^st^ metatarsal region. The first three sets of parameters are material properties, and the final set involves altering the frictional coefficient from 0.6.

**Figure 4:**
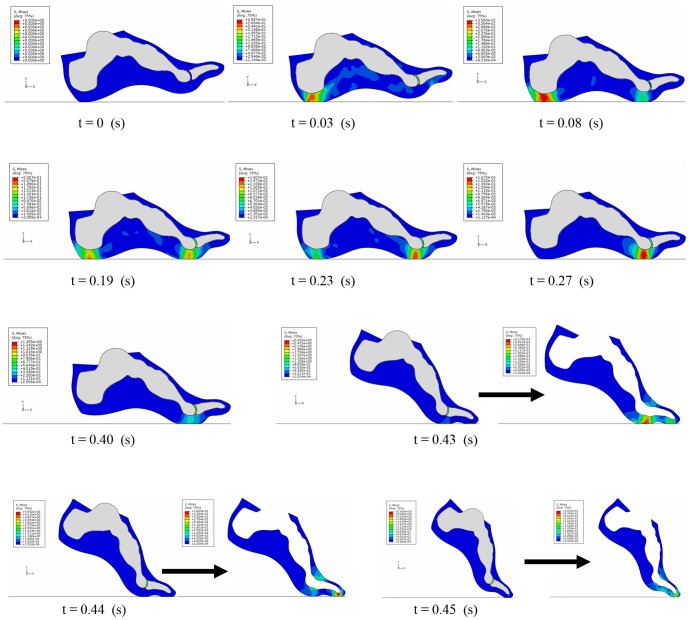
Predicted von Mises stress distribution at 10 representative instants of time over whole stance phase.

**Figure 5:**
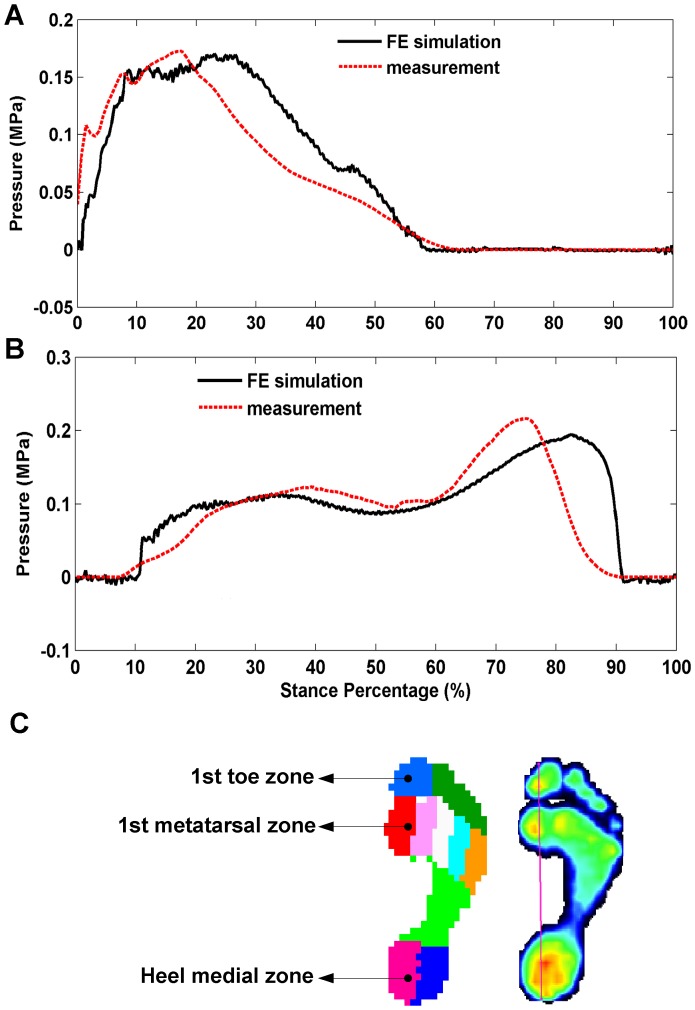
The calculated plantar pressure changes over the whole stance phase compared to the measurement data. (A) in heel medial region. (B) in the first metatarsal head region. The foot-ground contact area was divided into ten zones in the RSscan pressure plate system (C), so the zonal pressures could be directly calculated.

The predicted von Mises stress distributions on the foot at 10 typical instances of time over the stance phase are shown in Fig. 4. The location with the highest von Mises stress moved predominantly from the heel region to the toes over the stance phase. The peak plantar pressure changes at the heel medial and 1^st^ metatarsal regions over the whole stance phase are shown in Fig. 5. The FE simulation predicted peak plantar pressures of 0.17 MPa and 0.19 MPa at the heel medial and the 1^st^ metatarsal regions respectively, which are close to the peak pressure records (0.17 MPa and 0.21 MPa) measured by the pressure plate. However, there are some discrepancies on the timings of the peak pressures. The predicted peak plantar pressures at both the heel medial and 1^st^ metatarsal regions occurred about 10% of the stance phase later than in the measured data for walking.

Table 2 lists the RMSE and rRMSE results of the sensitivity analysis of foot material properties,the foot-ground frictional coefficient and the damping coefficient of the linear bulk viscosity. It can be seen that, for linearly elastic material, decreasing the Young's modulus of the foot soft tissues to 20% made no any particular improvement on the simulation results. While the prediction accuracy in horizontal ground reaction force and peak plantar pressures in the heel medial and 1^st^ metatarsal regions have been improved slightly by increasing the Young's modulus. Representing the material property of the soft tissue using either of Ogden or polynomial hyperelastic formulations did not lead to any improvement on simulation results compared to the linear elastic representation, except for the case of CoP for the polynomial hyperelastic representation, where the prediction error is decreased slightly compared to the baseline result using the linear elastic material property. Our sensitivity analysis of the frictional coefficient shows that the ground reaction force predictions are slightly improved by either increasing or decreasing the frictional coefficient. However, this leads to poor prediction in the foot and phalanx rotation angles, especially when decreasing the frictional coefficient. The RMSE and rRMSE results of the damping coefficient sensitivity analysis showed that the oscillation in the ground reaction forces can be significantly reduced by changing this parameter and the best prediction results are obtained when *b* = 6.0. The calculated peak plantar pressures at three representative instants of time in the gait cycle by using the dynamic FE simulation were compared in Table 3 with the results obtained by using the static FE simulation. In the early stance phase (at 0.03 s), the dynamic FE simulation predicted a peak plantar pressure of 0.134 MPa at the heel medial region, which is about 10% closer to the measured 0.14 MPa peak pressure than the static FE simulation. While in the middle stance, the quasi-static simulation provides slightly better prediction than the dynamic simulation in the heel medial region. However, in the 1^st^ metatarsal region, the dynamic FE simulation provided much better result than the static FE simulation in the middle stance, which is about 33% closer to the measured peak pressure data. In the late stance, both dynamic and static simulations predicted much higher peak plantar pressures than the measurement data.

**Table 3:** The calculated peak pressure in different foot contact regions using both the dynamic FE simulation and the quasi-static FE simulation compared to the recorded pressure plate data.

Time instant	Contact zone	Peak pressure (MPa)		
		Dynamic simulation	Quasi-static simulation	Measurement
0.03s	heel medial	0.13	0.12	0.14
	1^st^ metatarsal	–	–	–
	1^st^ toe	–	–	–
0.19s	heel medial	0.090	0.070	0.060
	1^st^ metatarsal	0.11	0.070	0.12
	1^st^ toe	–	–	–
0.44s	heel medial	–	–	–
	1^st^ metatarsal	0.010	0.010	0.00
	1^st^ toe	0.13	0.070	0.030

## Discussion

In this study, we have developed a subject-specific 2D FE foot model that dynamically simulates the mechanics of human foot structure during normal walking. Our dynamic simulation of the entire stance phase produced visually realistic results and a compelling match with measured ground reaction forces, foot rollover angle, phalanx rotation angle, CoP displacement and plantar pressure changes in most of the stance phase, indicating that the model is reasonably valid with some exceptions noted below.

The major discrepancies between our simulation results and measurement data can be explained by the limitations and assumptions of the model. Apparent fluctuations were observed in the predicted ground reaction forces especially in the horizontal component in the early stance phase just after heel-strike. These periodic vibrations are probably due to the foot-ground impact at heel-strike, which may be more prominent at a faster walking speed, as adopted in the representative gait trial used in this study. It appears that the large kinetic energy induced by the heel-strike impact could not be effectively absorbed and attenuated immediately after heel-strike as in actual human feet [35]. The material properties and frictional coefficient sensitivity analyses suggests that this problem could not be fully remedied by changing the Young's modulus of the linear elastic material, by representing the material properties using nonlinear hyperelastic models, or by varying the foot-ground frictional coefficient (see Table 2). This may be due to the simplified foot structure in the modelling. Another possible explanation is the numerical effect. A previous study on the foot/sock-insole interaction [25] using the same explicit algorithm produced the similar oscillation in the simulated shear force time histories. Our sensitivity analysis on the linear bulk viscosity defined in the integrator of the explicit solver showed that a substantial part of the oscillation is due to the numerical effect of the dynamic integration and a proper tuning of the linear bulk viscosity can significantly reduce the force fluctuations in both vertical and horizontal directions. It's noteworthy that there is still some small oscillations present in the horizontal force component after the linear bulk viscosity is tuned. This may be due to the contact algorithm used in the ABAQUS software to handle the horizontal contact mechanics. This strongly suggests that investigation is needed in the future to explore the effect of contact algorithms on model predictions.

Additionally, our simulations overestimated the CoP displacement in late stance (see Fig. 3D). This may be because the simulations predicted excessive toe rotations before toe-off, which made the phalangeal bones rotate around the toe tip in the late stance, thus resulting in a larger CoP displacement. In contrast, during normal human walking the phalangeal bones mainly stay in contact with the ground until toe-off. This discrepancy may be explained by the absence of ankle plantarflexor muscle activations in the model. The “windlass mechanism” induced by the ankle plantar-flexor contractions may help to stretch the foot plantar fascia [36] and hereby keep the toes in contact with the ground during late stance.

The FE model predicted foot rollover angles and phalanx rotation angles in the sagittal plane reasonably well, but with major discrepancies in the early stance phase (in the first 10% of the stance phase). This is probably due to the error in setting the initial angle values at heel strike. The gait measurement data were used to define the initial angles, which used the reflective markers attached on the subject's foot skin surface. In contrast, in the FE foot model, node points on the bones were used to define the rotation angles. Although our best effort was made to match the marker positions and node positions, some apparent differences still persist. This may be particularly difficult at heel strike due to the possible large skin movement artifacts induced by the foot-ground impact. X-ray cameras or bone-mounted motion analysis markers would be needed to reduce this error.

The peak plantar pressures from the FE model predictions compared well with the pressure plate measurement data at both heel medial and 1^st^ metatarsal regions, but with a time delay of about 10% stance phase. This may be due to the simplifications in the model construction. In this study, the complex 3D foot structure is considerably simplified as a 2D planar model extruded with a width to match the total foot mass. This may have lead to discrepancies in dynamic structural responses, which may have resulted in a phase shift in peak pressures. In addition, the lack of consideration of sesamoid bone in the modelling may lead to underestimation of the peak plantar pressure at the 1^st^ metatarsal region and may also cause some delay in the plantar pressure response. Moreover, the soft tissue material properties may also play a role in determining the plantar pressure distribution. In order to reduce the complexity of the explicit FE simulation, the soft tissues were modelled as homogeneous and isotropic elastic materials in this study, was an assumption adopted by most of the previous FE foot models [12]–[15], [18], [25], [27], [37], [38]. However, an experimental study [39] showed that the foot plantar soft tissue demonstrated strong anisotropic, time-dependent and viscoelastic material behavior. Inappropriate material property representations may lead to large errors in plantar pressure predictions, which are also suggested by the results of our sensitivity analysis of material properties.

In this study, the foot was allowed to move freely with respect to the ground without any predefined kinematic constraints. The foot-ground interaction was modelled as a kinematical contact with friction. This offers an alternative approach to modelling the dynamic foot-ground contact. It has the benefit that it also estimates the dynamic contact stress distribution, which cannot be obtained using the discrete spring [4] or uni-lateral rigid contact [8] methods. By incorporating these features into a forward dynamics musculoskeletal model, this foot model could also be used to conduct the simultaneous simulation of soft tissue mechanics and musculoskeletal dynamics [19], [20]. This could help alleviate a major problem of many gait simulations: unrealistic foot-substrate mechanical interactions and poor matches with experimental ground reaction force data.

Direct comparison of the results of our model with previous static or quasi-static FE foot models is difficult because appreciably different loading and boundary conditions have been used in the previous studies. However, some apparent differences are found in our analysis when comparing the results of the dynamic FE simulation with the results of the quasi-static simulation based on the same foot model described in this study. The prediction accuracy of the peak plantar pressures has been improved by 10% in the heel medial region in the early stance (at 6% of the stance phase, just after heel strike) and by 33% in the 1^st^ metatarsal region in the middle stance (at 40% of the stance phase, shortly after metatarsal head strike) respectively, by using the dynamic FE simulation. This strongly suggests that the dynamic formulation of the FE foot model may have some advantages over the quasi-static formulation in predicting the stress and strain conditions for dynamic motions especially when ground-foot impacts are involved. The small discrepancy produced in the late stance may be explained by the excessive toe rotation generated due to the lack of the modelling of individual ankle plantarflexor muscles.

Moreover, the close agreement of our predicted ground reaction forces, CoP displacement and foot bone motions with the individualized measurement data indicates that the dynamic FE model proposed in this study made an encouraging attempt towards a more realistic and robust approach for simulating human foot dynamics than the static or quasi-static methods. Our ongoing work involves more accurate representation of the soft tissues using non-linear isotropic viscoelastatic material, realistic anatomy of the complex 3D foot structures and modelling the contractions of individual muscle groups [40]. This would lead to a better understanding of the dynamic behaviors and the internal loading conditions of the foot musculoskeletal structures and thereby facilitate clinical diagnosis, footwear design and injury prevention.

## Conclusion

A subject-specific dynamic finite element foot model was constructed and tested in this study, which demonstrated the potential to provide a more rigorous method for simulating human foot dynamics than previous static or quasi-static FE simulations, and may lead to a better understanding of the structural dynamics of the human foot musculoskeletal system during normal and even pathological functioning. Our ongoing work involves the extension of the current FE foot model to a fully muscle-driven dynamic three dimensional FE musculoskeletal model with detailed representation of all major anatomical structures and also the investigation into the foot dynamics and structural responses during different motor activities.
