# Correction: A Dynamic Finite Element Analysis of Human Foot Complex in the Sagittal Plane during Level Walking

**DOI:** 10.1371/annotation/ce351166-09b8-4a6b-bf8a-a0858670c034

**Published:** 2014-01-03

**Authors:** Zhihui Qian, Lei Ren, Yun Ding, John R. Hutchinson, Luquan Ren

The corresponding author of the article was not correctly indicated. The correct corresponding author is not the last author, but instead the second author, Lei Ren. The corresponding author's email address is: lei.ren@manchester.ac.uk.

In addition, two grant numbers from the Key Project of the National Science Foundation of China were incorrectly listed in the Funding Statement. The Funding Statement should read: "This work was supported by the International Cooperation Project of National Natural Science Foundation of China (No. 50920105504), a Biotechnology and Biological Sciences Research Council Grant (No.BB/H002782/1), the Project of National Natural Science Foundation of China (No. 51105167), the Fundamental Research Founds of China (No. Z201105), the Key project of National Science Foundation of China (No, 51290290 & 51290292), the postdoctoral project (No, 2013M530985) and the scientific and technological development planning project of Jilin Province (No, 20130522187JH). The funders had no role in study design, data collection and analysis, decision to publish, or preparation of the manuscript." 

